# The Usability of Diabetes MAP: A Web-delivered Intervention for Improving Medication Adherence

**DOI:** 10.2196/humanfactors.5177

**Published:** 2016-05-12

**Authors:** Lyndsay A Nelson, Magaela C Bethune, Andrea E Lagotte, Chandra Y Osborn

**Affiliations:** ^1^ Department of Medicine Vanderbilt University Medical Center Nashville, TN United States; ^2^ Center for Health Behavior and Health Education Vanderbilt University Medical Center Nashville, TN United States; ^3^ Department of Human & Organizational Development Vanderbilt University Nashville, TN United States; ^4^ Department of Biomedical Informatics Vanderbilt University Medical Center Nashville, TN United States; ^5^ Center for Diabetes Translational Research Vanderbilt University Medical Center Nashville, TN United States

**Keywords:** Website, Usability Testing, Type 2 Diabetes Mellitus, Medication Adherence, Intervention

## Abstract

**Background:**

Web-delivered interventions are a feasible approach to health promotion. However, if a website is poorly designed, difficult to navigate, and has technical bugs, it will not be used as intended. Usability testing prior to evaluating a website’s benefits can identify barriers to user engagement and maximize future use.

**Objective:**

We developed a Web-delivered intervention called Diabetes Medication Adherence Promotion (Diabetes MAP) and used a mixed-methods approach to test its usability prior to evaluating its efficacy on medication adherence and glycemic control in a randomized controlled trial.

**Methods:**

We recruited English-speaking adults with type 2 diabetes mellitus (T2DM) from an academic medical center who were prescribed diabetes medications. A trained research assistant administered a baseline survey, collected medical record information, and instructed participants on how to access Diabetes MAP. Participants were asked to use the site independently for 2 weeks and to provide survey and/or focus group feedback on their experience. We analyzed survey data descriptively and qualitative data thematically to identify participants’ favorable and unfavorable experiences, characterize usability concerns, and solicit recommendations for improving Diabetes MAP.

**Results:**

Enrolled participants (N=32) were an average of 51.7 ± 11.8 years old, 66% (21/32) female, 60% (19/32) non-Hispanic White, 88% (28/32) had more than 12 years of education, half had household incomes over $50,000, and 78% (25/32) were privately insured. Average duration of diagnosed diabetes was 7.8 ± 6.3 years, average A1c was 7.4 ± 2.0, and 38% (12/32) were prescribed insulin. Of enrolled participants, 91% (29/32) provided survey and/or focus group feedback about Diabetes MAP. On the survey, participants agreed website information was clear and easy to understand, but in focus groups they reported navigational challenges and difficulty overcoming user errors (eg, entering data in an unspecified format). Participants also reported difficulty accessing the site and, once accessed, using all of its features. Participants recommended improving the site’s user interface to facilitate quick, efficient access to all features and content.

**Conclusions:**

Adults with T2DM rated the Diabetes MAP website favorably on surveys, but focus groups gave more in-depth feedback on the user experience (eg, difficulty accessing the site, maximizing all of the site’s features and content, and recovering from errors). Appropriate usability testing methods ensure Web-delivered interventions work as intended and any benefits are not diminished by usability challenges.

## Introduction

Among adults with type 2 diabetes (T2DM), approximately 1 in 3 do not take their medications as prescribed [1], and nonadherence is associated with suboptimal glycemic control [2], hospitalizations [3,4], and pre-mature death [4,5]. Very few interventions improve medication adherence, and among those that do, effects are generally small [6]. Moreover, most efficacious interventions have been delivered face-to-face, making them more labor-intensive and less feasible in busy clinic settings [7]. An estimated 84% of adults in the United States use the Internet [8], so the automated nature of Web-delivered interventions makes them a more feasible alternative to face-to-face approaches [9].

Web-delivered interventions have mixed effects on health behaviors [10-12] and varied effects on glycemic control [13,14]. Wide variability in both the time spent using websites and how they are used may explain their mixed effects on health behaviors and outcomes [15]. Website engagement varies widely between studies [16,17], and more engagement is often associated with greater improvement in outcomes [18,19]. A fundamental determinant of website engagement is a website’s usability [20], or how easy a user interface is to use.

The evaluation of a website’s usability is necessary before testing its potential efficacy on health behaviors and outcomes [21]. Website usability is the extent to which users can effectively, efficiently, and satisfactorily interact with a website [22]. Six factors determine a site’s usability, including (1) an intuitive design (ie, the site is easy to understand and navigate), (2) its ease of learning (ie, how quickly a user can learn basic site tasks), (3) its efficiency of use (ie, how quickly a user can complete site tasks), (4) its error frequency and severity (ie, how often users make errors, the seriousness of the errors, and how users recover from errors), (5) its memorability (ie, how well a user can remember the site to use it effectively in the future), and (6) its subjective satisfaction (ie, how much the user enjoys using the site) [22]. Usability testing focuses on measuring a website’s capacity to excel in each of these 6 areas.

Usability testing ensures a Web-delivered intervention works as intended, so the target audience uses it to the degree needed to reap its potential benefits [15]. Usability testing studies often employ quantitative surveys, but a qualitative approach can reveal more usability problems and concerns than surveys alone [21,23]. A mixed-methods approach includes both and provides a comprehensive assessment of website usability. Therefore, we used a mixed-methods approach focused on the 6 usability areas [22] to: (1) identify the favorable and unfavorable aspects of the Diabetes Medication Adherence Promotion (Diabetes MAP) website, including its usability challenges, and (2) solicit ideas for improving the site’s usability prior to evaluating its impact on medication adherence and glycemic control in a randomized controlled trial.

## Methods

### Diabetes MAP Intervention

**Figure 1:**
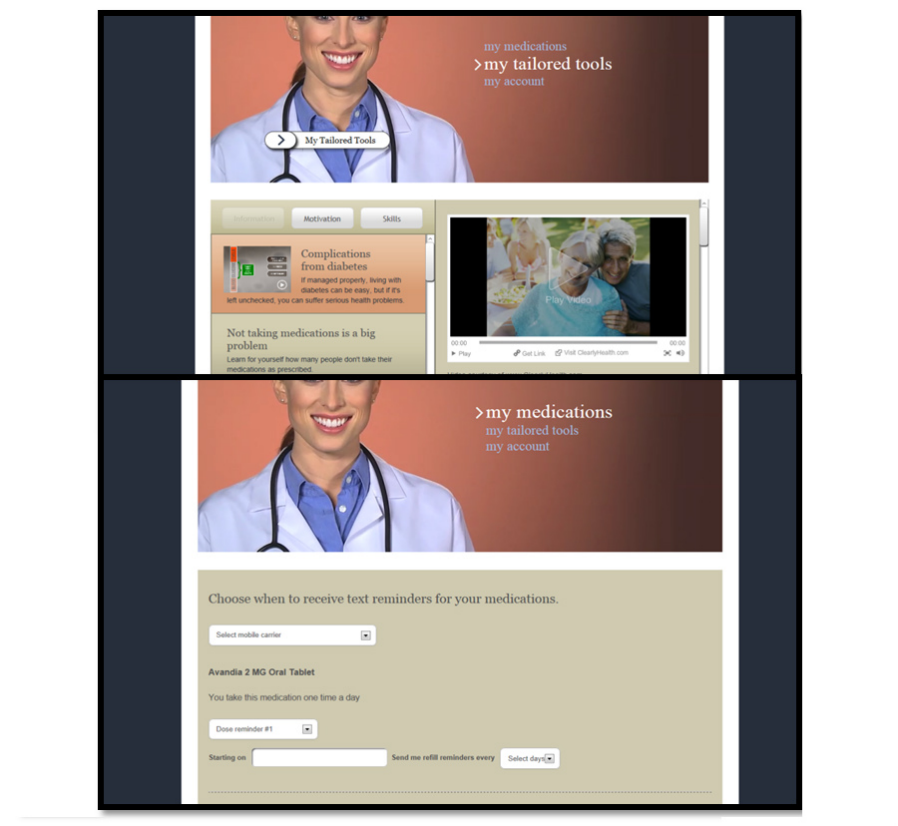
Top Panel: Diabetes MAP screenshot of the My Tailored Tools page presenting videos and content to address a user's barriers to adherence. Bottom Panel: Diabetes MAP screenshot of the page where a user can set up text message medication reminders.

Diabetes MAP is a self-guided, Web-delivered intervention designed to promote medication adherence among patients with T2DM. Diabetes MAP’s content is grounded in the Information-Motivation-Behavioral skills (IMB) model of medication adherence [24,25]. Studies in diabetes [24,25] and other chronic disease contexts (eg, HIV) [26] suggest a patient’s medication adherence depends on his or her adherence-related information, motivation to take medications, and adherence-related behavioral skills. Therefore, Diabetes MAP’s intervention content addresses user-specific barriers to adherence in each of these domains. Upon registering for an account and logging in to the Diabetes MAP site, users are asked to create a medication list by searching for RxNorm-generated medications. Next, they are asked a series of questions to assess their medication adherence-related information, motivation, and behavioral skills barriers. Entered medications and responses to these questions populate a separate page titled, My Tailored Tools (Figure 1, Top Panel). This page responds to a user’s early inputs (eg, medications entered, barriers to adherence) with a toolbox of tailored regimen-specific and IMB-model based intervention content.

My Tailored Tools houses 30 educational videos and 11 pieces of static content to enhance user-specific adherence-related information, motivation, and behavioral skills. Informational content is both medication class-specific (eg, a video on the key facts about metformin and how it works in the body for patients prescribed metformin, a video of how insulin works in the body for patients prescribed insulin) and conveys the importance of adherence for glycemic control and preventing complications (eg, a video showing the complications that can occur from not taking medications as prescribed). Motivational content is intended to enhance patients’ personal and social motivation for adherence (eg, a video on how to overcome one’s fear of needles, static content presenting strategies for soliciting social support for adherence). Finally, behavioral skills content provides practical “how to” advice to ensure successful adherence (eg, a video with step-by-step instruction on how to inject insulin, a video on how to store insulin).

The Diabetes MAP website has additional capabilities. Its features (ie, functionality built into the site to enhance the user experience) allow users to perform various tasks (ie, clearly defined assignments to complete within a website). For example, upon creating a medication list, users can print and email this list, learn about each medication listed, set up medication dosing and refill reminders sent as text messages to their mobile phone (Figure 1, Bottom Panel), and connect to a patient portal account (ie, My Health at Vanderbilt) to communicate with healthcare providers about medication side effects and prescription reauthorizations via secure messaging. As noted above, users can also complete an IMB model-based barriers-to-adherence assessment and view user-specific educational videos and content to address users’ IMB model-based barriers. Finally, the website includes navigational videos explaining the site’s features and giving instructions on how to complete tasks. Diabetes MAP was not designed for a specific user, but we made design choices to account for potential literacy, visual, and auditory limitations of all users. Such choices include presenting simplified language in large font, the option to watch and/or listen to videos or read video scripts, and a full-screen option to improve video visibility.

### Participants and Recruitment

To test the usability of the Diabetes MAP site, we recruited English-speaking adults from an academic medical center who were diagnosed with T2DM, prescribed diabetes medications, and had Internet access to participate in a mixed-methods study. Recruitment strategies included advertisements about the study, referrals from healthcare providers, medical center listserv announcements, and approaching patients waiting in an adult diabetes specialty clinic or adult primary care clinic. The total number of participants enrolled (N=32) reached the target enrollment for qualitative (at least 5) and quantitative (at least 20) usability testing [27,28]. The Institutional Review Board at Vanderbilt University Medical Center approved all study procedures prior to participant enrollment.

### Procedures

A trained research assistant (RA) scheduled interested and eligible participants to meet individually in a private room at the medical center. The RA administered (1) informed consent, (2) a survey by reading survey items and response options out loud or by distributing one available in paper-pencil format or electronic format via Research Data Capture (REDCap™) [29] that could be completed independently, and (3) a 1-page instruction guide on how to locate and access the Diabetes MAP website, which each participant was asked to independently use for 2 weeks. With permission, the RA also reviewed each participant’s medical record to collect clinical data.

We used Mouseflow ApS™ to measure participants’ use of Diabetes MAP. After 2 weeks, the RA invited participants to provide feedback on the site’s usability by completing a 20-minute survey and attending a 60-minute focus group session. The survey could be completed in REDCap™ via an email link or in-person immediately before the focus group session. A trained focus group facilitator used semistructured a priori questions to assess participants’ experiences with Diabetes MAP, demonstrate the site on a projector screen, and elicit responses and impressions of the site. This method is consistent with the pluralistic walkthrough approach to usability testing that involves stepping through a system with users to understand their perceptions of and experiences with a system [30,31]. The pluralistic walkthrough approach reveals users’ uncertainty with a system’s features and tasks better than other usability methods [31]. We asked participants if they had challenges with using the site, their most and least favorite aspects of the site, their perceived benefits of using the site, and any recommendations they had for improving it. All sessions were audio-recorded. Recordings were transcribed verbatim and de-identified prior to analyses. We compensated participants up to $155 for completing a survey at enrollment ($25), using Diabetes MAP ($8 per hour, up to 10 hours), completing the follow-up survey ($15), and participating in a focus group ($35).

### Measures

#### Demographic Characteristics

Demographic information included participants’ age, gender, race/ethnicity, years of education, annual household income, and health insurance status. We asked participants whether they owned a mobile phone and used text messaging with their phone to better understand participants’ reasons for setting up or not setting up medication dosing and refill text message reminders in Diabetes MAP.

#### Clinical Characteristics

Participants self-reported duration of diagnosed diabetes in years and months, and the number and type of diabetes medications prescribed, including insulin. The RA reviewed each participant’s medical record to confirm a T2DM diagnosis and the quantity and type of prescribed medications, and to collect participants’ most recent glycated hemoglobin A1c test result to characterize the sample’s glycemic control.

#### Website Usage

We assessed participants’ website usage with data logged by Mouseflow ApS™. Specifically, we assessed the total number of days users initiated a session by logging into Diabetes MAP, the total number of minutes users were logged into Diabetes MAP (ie, from the time they created an account until the study was over), and the average number of minutes logged in per days logged in. We also captured whether users set up text message reminders to take their medications or refill prescriptions.

#### Usability

We assessed Diabetes MAP’s usability with 10 items adapted from the Computer System Usability Questionnaire (CSUQ) [32]. Because the CSUQ assesses the subjective usability of a general computer system, we adapted its items to specify the subjective usability of Diabetes MAP. Example items include: “Overall, it was easy to learn to use Diabetes MAP” and “When I make a mistake in Diabetes MAP, I recover easily and quickly.” Response options ranged from 1 (strongly disagree) to 5 (strongly agree), with higher scores indicating more favorable usability ratings.

### Data Analyses

We used SPSS version 21.0 to summarize quantitative data using means and standard deviations (SD), or frequencies and percentages as appropriate. We used selective coding [33] to identify focus group comments and conversations [34] addressing the 6 areas of website usability: (1) intuitive design, (2) ease of learning, (3) efficiency of use, (4) error frequency and severity, (5) memorability, and (6) subjective satisfaction [22]. First, we read focus group transcripts in their entirety, highlighting participant comments related to opinions about, experiences with, and suggestions for Diabetes MAP. Next, we integrated similar comments into categories. After an iterative process of integration and refinement, we mapped categories of responses onto each usability area. Units of analysis included single participant comments and multi-participant conversations reporting similar or different experiences with the site and suggestions for improving the Diabetes MAP user experience (eg, strategic placement of instructions, features and Web content).

## Results

The sample (N=32) was on average 51.7 ± 11.8 years of age. Most were female (66%, 21/32), non-Hispanic White (NHW; 60%, 19/32), had at least some college education (88%, 28/32), and were privately insured (78%, 25/32); half had incomes above $50,000 (Table 1). The average HbA1c was 7.4% ± 2.0%, and 38% (12/32) were on insulin. Most participants (91%, 29/32) provided feedback about Diabetes MAP via survey and/or focus group participation (up to 5 participants per group). The characteristics of our sample reflect the characteristics of the academic medical center patient population in which they were recruited from [35]. The medical center patient population is predominately NHW (74%), well-educated (ie, over 90% having education beyond a high school degree), with relatively high incomes, and private insurance (93%) [35].

**Table 1:** Participant characteristics (N=32).

Characteristic		Mean ± SD or n (%)	Range
Age, y		51.7 ± 11.8	26.7-73.4
Female		21 (66)	
Race/ethnicity	White (non-Hispanic)	19 (60)	
	Black (non-Hispanic)	8 (25)	
	Hispanic	3 (9)	
	Asian	2 (6)	
Education, y		16.3 ± 2.8	12.0-24.0
Annual household income	Less than $14,999	3 (9)	
	$15,000 to $24,999	4 (13)	
	$25,000 to $49,999	9 (28)	
	$50,000 to $74,999	7 (22)	
	$75,000 or more	9 (28)	
Insurance status	Private insurance	25 (78)	
	TennCare/Medicare	6 (19)	
	No insurance	1 (3)	
Own a mobile phone		32 (100)	
Text message with phone		26 (81.3)	
Diabetes duration, y		7.8 ± 6.3	0.0-20.0
Number of diabetes medications		1.8 ± 0.8	1.0-4.0
Prescribed insulin		12 (38)	
A1c (n=31)^a^		7.4 ± 2.0	4.9-15.8

^a^One participant did not have an A1c test result in the medical record at the time of data collection.

Among all participants enrolled in the study, the average number of days users logged into the site was 4.2 ± 4.2 days during the 2-week period. The average number of hours logged into the site was 4.3 ± 4.8 hours, and the average time logged in per days logged in was 56.6 ± 47.2 minutes. Five participants (16%) set up text message reminders to take their medications and 4 participants (13%) set up text message reminders to refill their prescriptions.

### Quantitative Feedback

On the survey, participants rated Diabetes MAP’s usability above average (ie, scores of >3 on a 5-point scale) on each of the 10 items (Table 2). The total usability rating averaged across all items was 3.86 ± 0.90. We also mapped each survey item onto the usability area it most closely reflected. As shown in Table 2, participants rated the understandability of the site’s information (ie, intuitive design), the clarity of the site’s information (ie, intuitive design), and the pleasantness of interacting with the site (ie, subjective satisfaction) the most favorably. In contrast, participants rated the ease and quickness of recovering from mistakes (ie, error frequency and severity), the effectiveness of the site’s information in helping users complete tasks (ie, efficiency of use), and the ease of navigating the site (ie, intuitive design) the least favorably (Table 2).

### Qualitative Feedback: Usability Areas

Across 9 focus groups, participants shared experiences using Diabetes MAP, including concerns about its usability and recommendations for improvement. Of the 24 unique usability concerns reported, 14 concerns were mentioned in the first focus group and another 6 concerns were mentioned in the second focus group. By the fourth focus group, 95% of all unique usability concerns had been reported. Generally, participants’ experiences with Diabetes MAP were similar across age, gender, race/ethnicity, education, and income. Table 3 presents each unique concern organized by usability area [22] and the number of focus groups it was mentioned in. The 6 areas of website usability [22] provide a framework for examining participants’ usability concerns and recommendations for improvement.

**Table 2:** Survey items assessing Diabetes MAP’s usability, ranked most to least favorably.

	Related Usability Area	Respondents^a,b^, n	Mean ± SD
The information provided in Diabetes MAP is easy to understand.	Intuitive Design	28	4.3 ± 0.7
The information (such as help videos, on-screen messages, etc.) provided in Diabetes MAP is clear.	Intuitive Design	27	4.2 ± 0.8
My user interaction(s) with Diabetes MAP are pleasant.	Subjective Satisfaction	27	4.0 ± 0.9
Overall, it was easy to learn to use Diabetes MAP.	Ease of Learning	28	3.9 ± 1.0
Overall, I feel comfortable using Diabetes MAP.	Subjective Satisfaction	28	3.9 ± 1.0
It is easy to find the tools and information that I need.	Intuitive Design	27	3.8 ± 0.9
The organization of information in Diabetes MAP is clear.	Intuitive Design	27	3.8 ± 0.9
It is easy to navigate the Diabetes MAP website.	Intuitive Design	29	3.6 ± 0.9
The information provided in Diabetes MAP is effective in helping me complete tasks on the website.	Efficiency of Use	27	3.6 ± 0.9
When I make a mistake in Diabetes MAP, I recover easily and quickly.	Error Frequency and Severity	26	3.5 ± 0.9

^a^Number of participants providing a response for each item on a scale of 1 (strongly disagree) to 5 (strongly agree).

^b^Some participants indicated items were “Not Applicable” to their experience.

**Table 3:** Participants’ concerns with Diabetes MAP by usability area.

Usability Areas	Concern	Number of Focus Groups Reporting Concern
Intuitive Design	The site’s layout and content placement was confusing	4
	Unnecessary scrolling required to access site features and tasks	3
	Difficult to explore the site using the navigation menu	3
	Unclear how entering information into site tailored the user experience	1
	Unclear how to minimize navigational videos	1
	Location of navigational videos was confusing	1
Ease of learning	Instructions for accessing and using the site were unclear	4
	Directions for use within the site were unclear	3
	Navigational videos did not help with accessing features/completing tasks	3
	Navigational videos dysfunctional	1
Efficiency of Use	Unable to save progress with completing tasks	3
	Website pages took a long time to load	3
	Difficult to select time-zone using worldwide map	2
	Automatically logged out of site if stopping use for 20 minutes	2
	Website not compatible with other digital devices (eg, iPads®)	2
	Difficult to scroll through different site windows	1
Error Frequency and Severity	Website not compatible with different browsers	7
	Error messages encountered while trying to log in	6
	Difficult to search for medication names in medication list	3
	Difficult to search for medication doses in medication list	3
	Technical support was required to use website	1
Memorability	The site’s purpose was unclear	4
	Website URL was confusing and made accessing the site difficult	3
Subjective Satisfaction	The site had a non-user-friendly interface	2

#### Intuitive Design

An intuitively designed website is easy to navigate and understand [22]. When users understand a site’s layout and purpose, they can effortlessly explore it. The focus group facilitator demonstrated how all of Diabetes MAP’s features and tasks were intended to work, but some participants did not fully understand the site when they used it independently. For example, some participants said Diabetes MAP’s navigational videos were pleasant and helpful, but others said these videos distracted them from engaging with the most important aspects of the site. Some were unaware navigational videos were even available.

I didn’t even realize there was a video connected to [the image] until you pointed out that arrowhead.

62-year-old NHW male

In another instance, participants were unclear how information they entered into the site affected their user experience. For example, data entered at account creation (eg, entering one’s time zone and mobile phone number) impacts functionality elsewhere on the site (eg, receiving text message medication reminders in the appropriate time zone), and data entered into the IMB model-based barriers-to-adherence assessment impacts what videos and content are available for viewing in a user’s My Tailored Tools section of the site. As a result, some participants did not access or use certain parts of the site.

It was also common for participants to miss out on site features and functions entirely (eg, the option to print one’s medication list or set up text message reminders) because they were unable to locate them.

I am really frustrated because I would have loved [text message reminders]. I’m serious. Where was it?

55-year-old African American/Black female

Related to this issue were concerns with navigating between different types of content in Diabetes MAP. It was common for participants to describe difficulty reading task instructions, viewing educational videos, and using features on a single webpage. In 1 focus group, participants commiserated with one participant who said she could have used more assistance with exploring the site:

I [would have liked] more instructions to help navigate [the medication list] and clearly access the site.

26-year-old Hispanic female

Unintuitive design issues such as this one made it difficult for participants to successfully use and fully engage with Diabetes MAP.

#### Ease of Learning

Ease of learning refers to how fast new users of a website can learn and accomplish basic site tasks [22]. Focus group participants reported barriers to learning how to use Diabetes MAP, noting the site lacked clear, comprehensive instructions on how to perform certain tasks. When participants lacked the necessary information to accomplish basic tasks, they became frustrated.

[I] wasted a lot of time…. It had dragged on for 2 or 3 days when I could have actually been using [the site] and I had to contact you, which I didn’t really want to have to do. It was frustrating, to say the least, and I just felt like, “What’s wrong with me? What’s wrong with my computer?”

55-year-old African American/Black female

Other participants voiced confusion without frustration such as this participant who said the directions to enter one’s medications were confusing.

It wasn’t a huge challenge, but in the medication list, it didn’t specify if it wanted you to put in just your diabetes medicine or other medicines, so I put in all my medicines.... It would have been nice if it was more specific.

27-year-old NHW female

In the most extreme cases, some participants said the navigational videos did not help them, particularly when videos did not work. When asked about these videos, members of 1 focus group were united in their unsuccessful experience.

I never could get [the video] to play.

35-year-old NHW female

And I couldn’t either, and…I thought maybe it was my computer, but it wasn’t, it was [the videos] I guess.

47-year-old, African American/Black female

#### Efficiency of Use

Efficiency of use refers to how quickly a user can complete website tasks [22]. Some participants reported difficulty completing tasks in Diabetes MAP in a timely and efficient way. This was in part due to variability in website loading times on certain devices.

I felt like it was a little heavy to start with… iPads® can open it, but [it] needs a lot of time, even though I have high speed [Internet]. When you open it [on the] iPad®, you can’t get some clips unless you are [using] a desktop or laptop.

35-year-old NHW male

Other participants speculated loading delays were due to the size and volume of videos being streamed.

[The navigational and educational] videos take a lot of feed. It takes forever to load, and when you click [one], it doesn’t immediately work.

32-year-old NHW female

In some instances, participants were unable to save their progress on a task to revisit it and complete it later. The site also logs users out who are logged in, but who do not use the site for 20 minutes, which resulted in several participants losing task progress for partial completion.

If you do half of it, and you try to do something else, and the computer freezes or logs you out, you have to start all over again. Is there a possible way—I’m sure there is—to save it and come back to it to finish it?

55-year-old NHW male

Finally, some participants felt certain tasks were overly complicated and time-consuming. For example, the site asks users to select their time zone on a worldwide map instead of from a more efficient drop-down menu.

When it got to the time zone, and that little map came up…I was thinking if they had a drop down for [it]—it would probably be easier—instead of a map.

47-year-old African American/Black female

#### Error Frequency and Severity

Error frequency and severity refers to how often users make errors, the seriousness of the errors, and how users recover from errors [22]. Many participants encountered Web browser issues, made mistakes during task completion, and received error messages they did not understand or could not overcome. User errors began when first attempting to access the site. Despite receiving written instructions on the Web browser requirements for accessing Diabetes MAP, participants in 7 of 9 focus groups reported browser-related problems, and subsequent error messages. In some cases, this led participants to stop trying to access the site altogether.

It was really weird. I have multiple browsers of Internet Explorer®, and I kept trying to change them, thinking maybe I’m just not using the right compatibility thing. Finally, I was like, “OK, I’m just not going to look at this [website].”

26-year-old Hispanic female

Errors while creating and logging in to user accounts were mentioned in 6 out of 9 focus groups. To create an account, users are required to enter personal information using several entry methods including text fields and drop-down menus. If information is entered incorrectly, users receive error messages preventing further access. The recovery time needed to overcome these errors varied between participants. When participants were unable to access Diabetes MAP, some enlisted professional and nonprofessional technical support.

I just happened to know this computer guy who was coming in my [office] to do some other work and I asked him … I said, “Can you get this website up?” It took him a while, and this is all this man does is IT work.

55-year-old NHW female

Other participants reached out to study personnel who answered questions and provided remote assistance consistent with the written instructions participants were provided on how to access the site. Participants who encountered errors and did not seek assistance reported frustration and wasted time, causing some to give up using the site altogether.

#### Memorability

A website with memorability is one users can remember well enough to use it effectively in the future [22]. The memorability of Diabetes MAP was primarily hindered by its confusing URL. The Web address was lengthy, unintuitive, and included different types of punctuation and acronyms. Several participants mentioned difficulty with accessing the site specifically because of the URL. It was common for participants who forgot or mistyped the URL to search for the words “Diabetes MAP” in a search engine or in the search bar on the medical center’s homepage. These troubleshooting techniques led users to incorrect websites and information. For example, participants who searched “Diabetes MAP” within search engines were often misdirected, leading some to online geographic maps of diabetes treatment facilities rather than the intended website intervention. Similar issues occurred when searching for the site on the medical center’s homepage, as described by this participant:

I searched for Diabetes MAP on the medical center’s site and got directions for how to get to the Diabetes Center.

55-year-old NHW male

Despite being both told about the website’s intent and receiving an instructional handout with this information, participants in nearly half of the focus group sessions felt the website’s purpose was confusing. In some cases participants forgot Diabetes MAP’s purpose altogether, which led to using the website in unintended and ineffective ways.

I didn’t even realize it was just for taking medications until we came to this focus group.

55-year-old NHW female

#### Subjective Satisfaction

Subjective satisfaction is determined by how much the user enjoys using the website [22]. While discussing participants’ overall experience with using Diabetes MAP, a few participants mentioned concerns with the site’s user interface. These participants said Diabetes MAP was difficult to operate and understand, and therefore unenjoyable to use. One participant expressed his frustration with Diabetes MAP’s user interface:

I’m not an IT person, but I’m a supervisor in my department, and I do not have a problem [with computers].... But this one over here, it was like going against a brick wall, OK? It was not user-friendly, whatsoever.

55-year-old NHW male

It is important to note that subjective satisfaction concerns were limited and mentioned in only 2 of the 9 focus groups. Participants who successfully accessed the site’s features and tasks enjoyed it. Participants across focus groups highlighted several positive aspects of the website:

[Diabetes MAP] reinforced some of the things I knew, but also gave me some new information, so I thought that was very good—I really enjoyed the educational features. I thought they were very helpful, [such as] what [medications do] to your body [and] how to take your medications.

55-year-old African American/Black female

I liked the skills [section], where it showed those 4 people and their tips on when to take the medicine.

61-year-old African American/Black female

I liked the My [Tailored] Tools part—I liked being able to read about you know, the consequences of not taking care of yourself when you have diabetes…they had some tips that I found…helpful. I love the text message notifications—that has increased my compliance.

27-year-old NHW female

### Participant Recommendations

When participants voiced concerns about Diabetes MAP, they also gave suggestions for improving it. Common across all suggestions was a request for more simplicity and flexibility within the site. To improve the site’s ease of learning and efficiency of use, participants wanted more straightforward methods for accessing the site’s components. They suggested strategically placing instructions, features, and Web content in easily recognizable ways. Some participants wanted specific features to be accessible on every page, or clearly designated on their own page. For example, participants liked the idea of a page dedicated to setting up text message reminders. On this page, previously entered medication information (ie, name, dosage) would appear and users could set up reminders for when to take specific medications and order prescription refills.

To improve the site’s intuitive design, participants recommended the navigational videos be minimized or eliminated entirely.

[The navigational video] is a distraction for me because if I have to scroll down for whatever I have to do, it would be better for me if the video came up once you click it, and is then minimized.

43-year-old Asian male

Additionally, in reference to the site’s memorability, participants recommended using a simple and recognizable URL that is easy to locate with an online search. They also wanted Diabetes MAP’s purpose to be clear while using it (eg, spelling out the Diabetes MAP acronym and including images of diabetes medications throughout the website).

Participants wanted a simplified, streamlined Diabetes MAP user experience. In order to improve the site’s error frequency and severity, participants recommended increasing compatibility across multiple browsers, including older versions of commonly used Web browsers. Additionally, they requested the ability to use Diabetes MAP across multiple digital devices without loading time delays. They also stressed the importance of clear and accessible resources for user support (eg, the ability to contact study staff directly if they had issues or questions, an accessible and searchable help resource on the website itself). Finally, participants wanted functional, useful, and easy to recall navigational videos to further facilitate learning how to use the site.

Those who reported some dissatisfaction, but generally endorsed the utility of Diabetes MAP, felt it might be more appropriate for certain types of patients with diabetes. For example, some users diagnosed with diabetes for a longer period of time felt the website might be particularly helpful for newly diagnosed patients. Other users suggested the website might be more useful for younger, more technology-proficient patients who prefer technology-delivered information as opposed to more traditional print materials.

## Discussion

### Principal Results

Usability testing is issue-focused and designed to assess the extent to which users can easily, efficiently, and effectively perform tasks with a technical system. We employed a mixed-methods approach to understand the challenges of using a Web-delivered medication adherence promotion intervention called Diabetes MAP. Participants with diabetes provided ratings and descriptions of their experiences using the website, as well as recommendations for improving it. On surveys, participants agreed Diabetes MAP was helpful and easy to use, but, in focus groups, they mentioned 24 unique user concerns related to each of the 6 factors determining website usability [22].

Our quantitative results are comparable to other usability studies employing the CSUQ, in that total ratings were above average [36,37]. When comparing survey items rated most to least favorably with focus group comments, there were instances when survey ratings and quotes were discordant and concordant. For example, participants rated the understandability and clarity of the site’s information and the pleasantness of interacting with the site most favorably on surveys, yet, in focus groups, several participants expressed frustration with understanding how to complete tasks and navigate the website (ie, issues with the site’s ease of learning and intuitive design). Alternatively, many positive statements about the value of the site’s information and features (ie, subjective satisfaction) support these high ratings. Participants rated the ease and quickness of recovering from mistakes least favorably on surveys; focus group comments about error frequency and severity align with this low rating. In reconciling these inconsistencies and consistencies, it appears that while some participants had issues with understanding, navigating, and accessing Diabetes MAP, participants who successfully accessed the site, said it was enjoyable and helpful.

Recent usability studies of Web-delivered interventions for T2DM self- management yield results comparable to ours. In their evaluation of a Web-based dietary intervention, Ramadas et al found positive ratings of a website’s usability based on survey items; however, this study did not use qualitative assessments [38]. Alternatively, Yu et al used focus groups to examine the usability of their self-management website [39] and identified several of the same usability concerns we did with Diabetes MAP. Namely, participants mentioned issues with the website layout and organization, navigation, data entry, and language [39]. Although these issues can be applied to Web-delivered interventions generally, usability testing also reveals issues specific to a certain website [39].

Our research highlights the value of using mixed methods for usability testing. Had we relied on only survey data, we would have incomplete information on Diabetes MAP’s usability. Collecting qualitative data as part of usability testing reveals insights on unanticipated challenges and ideas for improving a site [21]. Additionally, involving members from the target audience is critical to understanding any unique needs of users for whom the site is intended [40]. The total time logged into Diabetes MAP during the 2-week period varied considerably across users*.* In a similar usability study, Heinrich et al had participants use a diabetes education site for 2 weeks, and participants visited the site an average of 3.6 ± 2.7 times and spent an average of 58.0 ± 56.1 total minutes on the site [41]. Compensation for time spent on the site was not reported. In our study, the more time spent using Diabetes MAP may reflect compensating participants per hour of use. Despite this, our qualitative results suggest some participants were discouraged from logging in more often because of the usability issues they encountered.

The US Department of Health and Human Services (HHS) has set forth peer-reviewed guidelines for improving the design and usability of websites [42]. Taking into consideration these guidelines and the results of our usability study, we identified key principles for website creation to promote an optimal user experience. First, it is crucial to employ both simplicity and clarity in Web design. Diabetes MAP users were often confused about how to complete tasks and navigate the site both because they encountered user errors and the layout was unintuitive. HHS suggests standardizing tasks to be performed in a similar way so tasks can be reliably repeated [42]. When requesting users to enter information, a standard entry format should be used across tasks (eg, drop-down boxes). Additionally, to account for working memory limitations, content from 1 page that need be remembered on other pages should carry over to those pages [42]. Finally, using simplified and familiar terminology for a URL and website features will minimize user confusion and frustration.

Our second principle is to design websites with the goal of keeping users informed and aware of website processes. As a general observation from our focus groups, users became frustrated with unanticipated incidents (eg, long downloading times, automatically being logged out). In some cases, it may not be possible to reduce the size of a page to minimize the time it takes for a webpage to load [42]. However, a website can notify users of the time required to download an image and/or supply progress indicators (eg, an hourglass) to communicate a waiting period and its duration [42]. In either case, the user expects additional time instead of wondering how long to wait. HHS also recommends warning users if a page is going to “time out,” so they can request extra time if needed [42]. Websites should also provide assistance to users who need additional help and ensure users are aware of this assistance. Resources should be easily accessible on the site, such as links providing more information about site content and a section for frequently asked questions [42].

### Limitations

There are limitations to our study. Because we recruited our sample from a single academic medical center, our findings may not generalize to other patient populations. However, our sample characteristics map onto the academic medical center’s patient population for whom Diabetes MAP was designed for. Additionally, although we were able to track time spent using Diabetes MAP, we were unable to track how participants used their Vanderbilt patient portal account because the two websites are not integrated. Considering the recent advancement of patient-provider communication in Web-delivered interventions, it will be valuable to track usage with this type of feature in future studies*.* Another limitation is the reliance on retrospective self-reports of users’ experiences with Diabetes MAP. Furthermore, the range from when a participant used Diabetes MAP to when he/she participated in a focus group session was 14-88 days. Other usability testing methods, such as think-aloud protocols, cognitive walk-throughs, and remote user testing facilitate real-time data collection of user interactions with a system [30]. No single usability evaluation method can capture all usability problems [43]. While think-aloud studies combat the limitations of retrospective studies, they are limited by an unnatural situation in which users may feel uncomfortable talking to themselves and the possibility many statements will be filtered (ie, not reflective of users’ actual experience) [44]. Although user feedback in our study was retrospective and the time between participants’ website use and focus group feedback ranged widely, we presented Diabetes MAP on a large projector screen and re-oriented participants to its pages, functions, and features to solicit real-time feedback and prompt recall of users’ experience. This method is similar to the pluralistic walkthrough approach that reveals users’ uncertainty with a system’s features and tasks better than other usability methods [31]. Additionally, the number of participants in each focus group allowed for the majority (over 80%) of concerns to be reported after only 2 focus group sessions. Moreover, the use of a mixed-methods approach provided a comprehensive evaluation of Diabetes MAP’s usability. Finally, because usability feedback is system-dependent and the current study is based on a specific website, our findings may not generalize to other Web-delivered interventions.

### Conclusions

Our findings highlight the importance of evaluating a website’s usability prior to testing its efficacy. For Web-delivered interventions to be used as intended, researchers, Web designers, and developers must plan sufficient time to perform usability testing [45]. Ideally, they should begin with design thinking to allow for experimentation, creation and prototyping, and feedback and redesign prior to final product presentation [46]. An understanding of the target group’s needs paired with iterative idea generation and development helps ensure the final website appeals to users and supports their using it [46]. Usability evaluation methods, including user-driven approaches, ensure users can optimally engage with and benefit from Web-delivered interventions [15,47]. Without ensuring a website’s usability, critical design and functionality issues may go unnoticed and unaddressed, thereby preventing a site’s benefits from being realized [15].

## Abbreviations

A1c: hemoglobin A1c
CSUQ: Computer System Usability Questionnaire
Diabetes MAP: Diabetes Medication Adherence Promotion
IMB: Information-Motivation-Behavioral skills
HbA1c: glycated hemoglobin A1c
HHS: US Department of Health and Human Services
RA: research assistant
REDCap: Research Data Capture
SD: standard deviation
T2DM: type 2 diabetes mellitus
NHW: non-Hispanic White
